# Biological network motif detection and evaluation

**DOI:** 10.1186/1752-0509-5-S3-S5

**Published:** 2011-12-23

**Authors:** Wooyoung Kim, Min Li, Jianxin Wang, Yi Pan

**Affiliations:** 1 Department of Computer Science, Georgia State University, Atlanta, USA; 2 School of Information Science and Engineering, Central South University, Changsha 410083, P. R. China

## Abstract

**Background:**

Molecular level of biological data can be constructed into system level of data as biological networks. Network motifs are defined as over-represented small connected subgraphs in networks and they have been used for many biological applications. Since network motif discovery involves computationally challenging processes, previous algorithms have focused on computational efficiency. However, we believe that the biological quality of network motifs is also very important.

**Results:**

We define *biological network motifs *as biologically significant subgraphs and traditional network motifs are differentiated as structural network motifs in this paper. We develop five algorithms, namely, EDGEGO-BNM, EDGEBETWEENNESS-BNM, NMF-BNM, NMFGO-BNM and VOLTAGE-BNM, for efficient detection of biological network motifs, and introduce several evaluation measures including *motifs included in complex, motifs included in functional module *and *GO term clustering score *in this paper. Experimental results show that EDGEGO-BNM and EDGEBETWEENNESS-BNM perform better than existing algorithms and all of our algorithms are applicable to find structural network motifs as well.

**Conclusion:**

We provide new approaches to finding network motifs in biological networks. Our algorithms efficiently detect biological network motifs and further improve existing algorithms to find high quality structural network motifs, which would be impossible using existing algorithms. The performances of the algorithms are compared based on our new evaluation measures in biological contexts. We believe that our work gives some guidelines of network motifs research for the biological networks.

## Background

Systems biology focuses on the study of complex interactions in biological systems, rather than the study of individual molecules such as DNA, RNA, proteins and metabolites [1]. One of the goals of systems biology is understanding the structures of all molecules and their interactions in a system level. Therefore major challenges are understanding the dynamic structures of small molecules and determining their functions in a living cell. Various types of biological interactions have been expressed in networks, which include transcriptional regulatory networks, signaling pathways, metabolic networks and protein-protein interaction (PPI) networks. Biological networks share some of structural properties of other complex networks, or have specific features of scale-free and small-world effect [2]. However, the properties have been questioned by Lacroix et al. [3] with a number of reasons including the incompleteness of networks and inconsistent link generation for the graphs. Therefore, the analysis extends to other network properties such as network clusters and network motifs.

As biological networks are massive and the size is still increasing, dividing the network into a number of clusters helps reveal specific local properties. Network motif, as another concept describing local properties of a network, is defined as a small connected subgraph appearing frequently and uniquely in a network. Similar to a protein sequence motif, network motif is defined as a over-repeated pattern, but it requires much more computation as the process involves isomorphic testing and repeated processes for uniqueness determination. Network alignment [4] and network querying [5] are analogous to network motifs, but while network motifs are defined with only structural information, network alignment and network querying require both of the topological and biological information. Previous network motif discovery algorithms include exact counting and approximation algorithms: Exhaustive recursive search (ERS) [6], enumerate subgraphs (ESU) [7] and compact topological motifs [8] are exact counting algorithms. For efficient detection, several approximation algorithms have been provided including edge sampling (MFINDER) [6], randomized version of ESU from a search tree (RAND-ESU) [9], and tree-filtering search which is NEMOFINDER[10]. Furthermore, parallel search algorithms have been developed to realize feasible exact counting algorithms [11,12].

Network motifs are used for many applications in biological networks. Feed-forward-loop (FFL) and bifan network motifs are identified as the typical patterns in different types of biological networks [13,14]. Przulj et al. [15] used network motifs as a relative graphlet frequency distance to distinguish different protein-protein interaction networks. Also motif frequencies are exploited as classifiers for network model selection [16]. Milo et al. [17] studied that networks of different biological and technological domains have been classified into different superfamilies on the basis of motif significance profiles. To predict protein-protein interactions, Albert I. and Albert R. [18] used network motifs successfully. In the study by Conant and Wagner [19], network motifs in transcriptional regulatory networks are not evolutionary conserved while network motifs in PPI networks are evolutionary related. On the other hand, network motifs are extended to 'motif modes' each of which has a certain topology and a specific functional property [20].

Through a number of network motif applications, however, we notice several problems regarding the biological meanings of network motifs, on top of the computational challenge for the detection. First, the biological quality of network motifs are not validated thoroughly. A network motif is selected only by its structural uniqueness and just small number of instances of the type are biologically exemplified. Second, only small portion of network motif instances are used for applications and others are ignored. Third, non-motifs, that is, structurally insignificant subgraphs, have not been analyzed in any studies, which are filtered out before applying to any applications. Fourth, it is still questionable what the network motifs really represent in biological networks.

As we believe that the biological quality of network motifs are also significant, we define a ***biological network motif ***in this paper. Throughout this paper, we refer a network motif as a **structural network motif **to distinguish it from a biological network motif. Unlike structural network motifs, biological network motifs are biologically significant small connected subgraphs regardless of the structure. The biological significance is unspecified in the definition, as it will be assigned flexibly by a goal of the application. We introduce EDGEGO-BNM, EDGEBETWEENNESS-BNM, NMF-BNM, NMFGO-BNM and VOLTAGE-BNM algorithms for efficient discovery of biological network motifs, and design new evaluation measures named, 'motifs included in complex', 'motifs included in functional module' and 'GO term clustering score'. Our algorithms compete with existing algorithms including ESU, RAND-ESU and MFINDER, and the performance are compared based on the new measures introduced in this paper. The main idea for our algorithms is to reduce the number of subgraphs to search by removing a number of edges from the original network and, at the same time, increase the discovery rate for biological network motifs. Experimental results with a couple of S. cerevisiae PPI networks demonstrate that EDGEGO-BNM and EDGEBETWEENNESS-BNM algorithms perform better than other algorithms in most of the measures. In addition, we show that all of our algorithms are applicable to the discovery of structural network motifs as well.

The work has three contributions to the study of network motifs: 1)We question biological meanings of network motifs which have not been focused by existing detection algorithms. New motif search algorithms and evaluation measures are developed based on these questions. 2)We design several algorithms combining the topological and biological information in a network. The algorithms further enrich existing algorithms in a biological context. 3)We develop a number of evaluation measures which qualify biological importance of network motifs. As we know of, this is the first time to suggest systematical evaluation measures for network motifs. With these contributions, we hope that our work gives some guidelines for the researches of network motifs in biological networks.

## Results and Discussion

In this paper, we define biological network motifs as biological meaningful network motifs and develop EDGEGO-BNM,EDGEBETWEENNESS-BNM, NMF-BNM, NMFGO-BNM and VOLTAGE-BNM algorithms for an efficient detection of biological network motifs. For clarification, traditional network motifs are referred as structural network motifs throughout this paper. The performance of each algorithm is compared based on three evaluation measures such as 'motifs included in complex', 'motifs included in functional module', 'GO (Gene ontology) term clustering score' which we design to assess biological quality of network motifs. Detail description of algorithms and evaluation measures are described in the "Methods."

### Data sets

**Figure 1:**
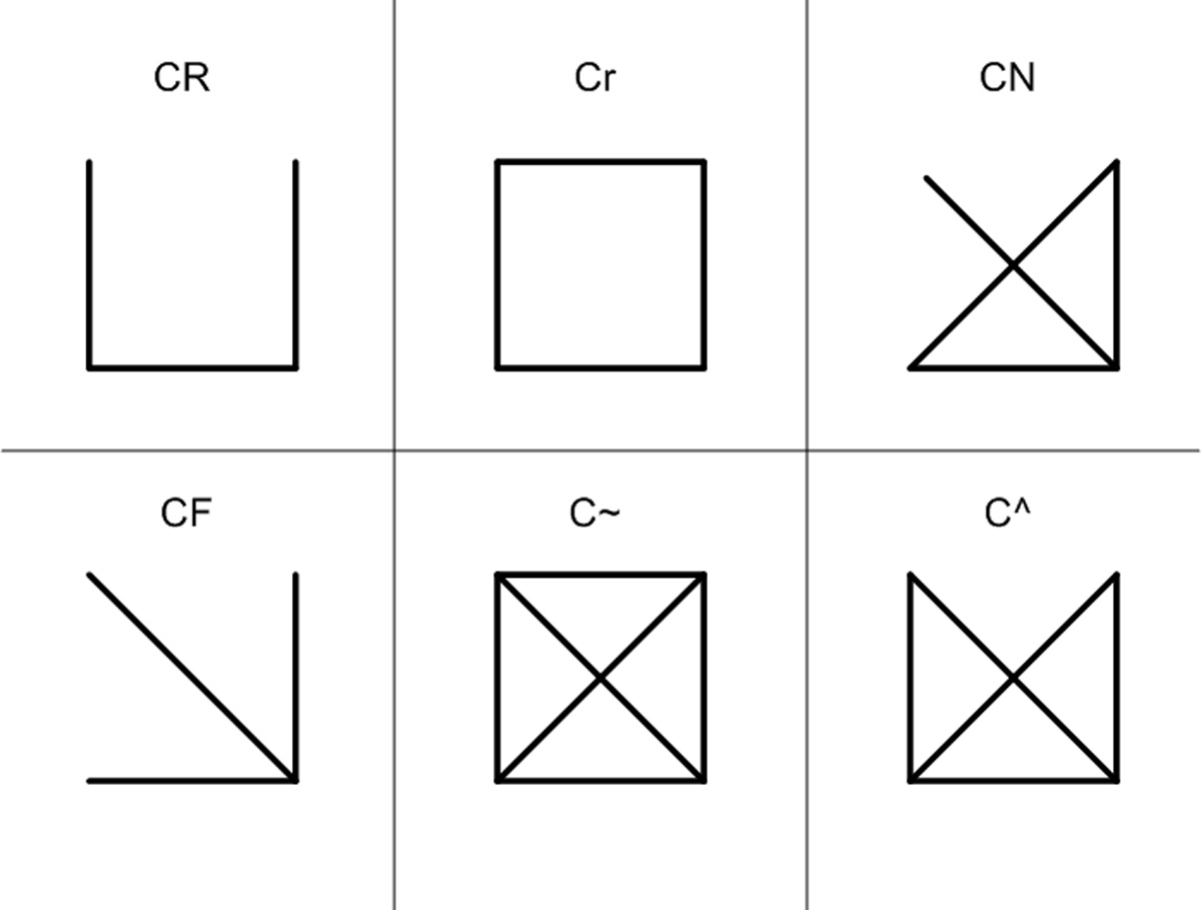
**Shapes and labels for 4-node subgraphs in an undirected network**. There are six types for 4-node subgraph in an undirected network. Each type is labeled with *Nauty *as shown as a text accordingly.

We test the performance of each algorithm with a couple of **PPI **of S. cerevisiae (yeast). We download a yeast core data, referred to 'Scere20101010' from **DIP **database [21] which has 2,130 proteins and 4,434 interactions and call this as **DIP Core **network. A network of 988 proteins and 2,455 with high confidence level of interactions, introduced as a high-throughput data in [22] and obtained from the authors of [23], is also used in this experiment. As it was conventionally referred to Y2k, it is called **Y2k **network. Since the increase of network motif size boosts the computational time and the number of motifs exponentially, we set the size of subgraphs as four to five for practical experiments. There are 6 types of isomorphic graphs for undirected 4-node subgraphs and 21 types for undirected 5-node subgraphs. Undirected 4-node subgraph types are labeled using Nauty program [24] as appeared in Figure 1.

### Comparison of the algorithms against different evaluation measures

We first enumerate all subgraphs of size four or five with ESU algorithm [7] and evaluate them with the evaluation measures introduced in this paper and name the experiment as an ESU. Then we run EDGEGO-BNM, EDGEBETWEENNESS-BNM, NMF-BNM, NMFGO-BNM and VOLTAGE-BNM algorithms and measure them with the same evaluation measures. Furthermore, we add experiments with two existing approximation algorithms; RAND-ESU and MFINDER. RAND-ESU searches subgraphs in a tree structure and it skips over some of the branches during its search. MFINDER randomly picks edges until it reaches the desired number of subgraphs. ESU algorithm enumerates all subgraphs and all other algorithms produce roughly 30% of total subgraphs by adjusting parameters. Additionally, we run FANMOD [9], which is a software implementing ESU, and investigate the topological properties for each type of subgraph in order to observe the relationships between biological network motifs and structural network motifs. Table 1 compares the performances of 8 different algorithms for 4-node biological network motifs from DIP core network, accessed by the following biological measures;'motifs included in complex', 'motifs included in functional module' and 'GO term clustering scores for BP, MF and CC.' The results of ESU, RAND-ESU and MFINDER algorithms are also provided as well for comparison purpose. The best result for each measure is marked as bolded in the table. EDGEBETWEENNESS-BNM algorithm provides highest rates for 'motifs included in complex' measure, but EDGEGO-BNM algorithm produces overall the best values compared to others. It is reasonable for the EDGEGO-BNM and NMFGO-BNM algorithms have good scores for GO term clustering score measures as they include GO term information. However, it is interesting to see that EDGEBETWEENNESS-BNM algorithm provides relatively good scores for all of the evaluation measures when this algorithm considers only topological property of the network. This suggests that the structural property helps infer meaningful biological information as well. We provide the results with 5-node biological network motifs as well in Table 2. Similar to the results in Table 1, EDGEBETWEENNES-BNM algorithm is the best for the 'motifs included in complex' term and EDGEGO-BNM is best for the rest of measures.

**Table 1:** Results of 4-node biological network motifs in the *DIP Core *network

Algorithm	Motif included in		GO Clustering score		
	
	Complex	Function	BP	MF	CC
ESU	.13	.205	.64	.51	.61
RAND-ESU	.13	.208	.65	.28	.46
MFINDER	.15	.299	.74	.57	.71
EDGEGO-BNM	.21	**.479**	**.85**	**.70**	**.80**
EDGEBETWEENNESS-BNM	**.28**	.392	.78	.60	.79
NMFGO-BNM	.18	.360	.78	.61	.75
NMF-BNM	.15	.230	.68	.54	.64
VOLTAGE-BNM	.26	.330	.77	.59	.75

EDGEBETWEENNESS-BNM performs best in 'motif included in complex' measure while EDGEGO-BNM performs best in other measures.

**Table 2:** Results of 5-node biological network motifs in the *DIP *Core network

Algorithm	Motif included in		GO Clustering score		
	
	Complex	Function	BP	MF	CC
ESU	.07	.097	.67	.51	.63
RAND-ESU	.07	.096	.66	.52	.62
MFINDER	.09	.167	.75	.56	.72
EDGEGO-BNM	.08	**.240**	**.87**	**.70**	**.79**
EDGEBETWEENNESS-BNM	**.14**	.210	.81	.59	.76
NMFGO-BNM	.08	.169	.71	.59	.60
NMF-BNM	.13	.104	.65	.53	.61
VOLTAGE-BNM	.08	.121	.71	.50	.67

EDGEBETWEENNESS-BNM performs best in 'motif included in complex' measure while EDGEGO-BNM pe forms best in other measures.

To see if the results are consistent with other network, we search biological network motifs in the **Y2k **network as well. The results are shown in Table 3 of 4-node subgraph and Table 4 of 5-node subgraph. Consistent with DIP core network, EDGEGO-BNM algorithm provides overall good scores except 'motifs included in complex' term and 'MF GO term clustering score'. EdGEBETWEENNESS-BNM algorithm is superior for the 'motifs included in complex' term too. It is interesting to see that NMFGO-BNM shows good scores as well in the Y2k network, which is because that NMF tends to produce better results with smaller data set. It is also appealing that the random-edge-selection algorithm (MFINDER) beats the random-vertex-select algorithm (RAND-ESU). This implies that edges are more important aspect for explaining its biological meanings.

**Table 3:** Results of 4-node biological network motifs in the *Y2k *network

Algorithm	Motif included in		GO Clustering score		
	
	Complex	function	BP	MF	CC
ESU	.501	.152	.61	.21	.67
RAND-ESU	.491	.126	.61	.23	.65
MFINDER	.586	.180	.65	.26	.72
EDGEGO-BNM	.603	**.463**	**.94**	.25	**.90**
EDGEBETWEENNESS-BNM	**.904**	.178	.82	.19	.84
NMFGO-BNM	.609	.434	.92	**.27**	**.90**
NMF-BNM	.819	.177	.76	.26	.80
VOLTAGE-BNM	.638	.200	.63	.26	.77

EDGEBETWEENNESS-BNM performs best in 'motif included in complex' measure. NMFBO-bnm performs best on 'MF' and 'CC clustering score' measures. EDGEGO-BNM performs best in the 'motif included in functional module measure 'BP, CC clustering score' measures. However all the algorithms perform poorly in 'MF clustering score' measure, with less than 30.

**Table 4:** Results of 5-node biological network motifs in the *Y2k *network

Algorithm	Motif included in		GO Clustering score		
	
	Complex	function	BP	MF	CC
ESU	.281	.083	.69	.17	.76
RAND-ESU	.305	.090	.71	.17	.77
MFINDER	.431	.096	.73	.21	.80
EDGEGO-BNM	.362	**.376**	**.99**	**.24**	**.96**
EDGEBETWEENNESS-BNM	**.814**	.087	.89	.13	.91
NMFGO-BNM	.445	.257	.98	.18	.96
NMF-BNM	.643	.073	.80	.18	.83
VOLTAGE-BNM	.665	.089	.82	.19	.85

EDGEBETWEENNESS-BNM performs best in 'motif included in complex' measure while EDGEGO-BNM performs best in other measures.

### Relationship between biological and structural network motifs

**Figure 2:**
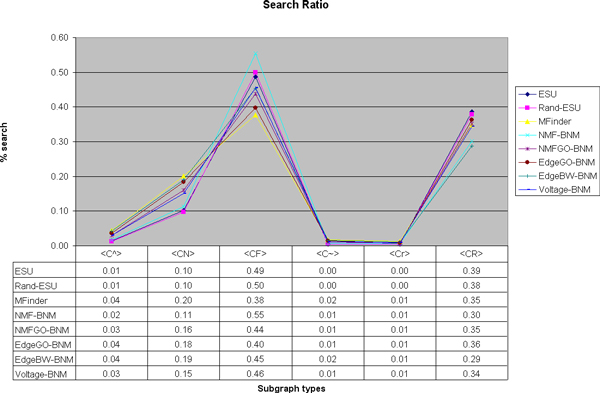
**DIP Core network: Search ratios based on the subgraph type**. The ratio of frequency of each type is relatively preserved and it indicates that our algorithms can be used for the structural network motif discovery as well. Relative frequencies of each algorithm is plotted with different colors of line. The horizontal axis indicated each subgraph type for 4-node subgraphs. The vertical axis shows the relative frequency of each type. The values are shown in the table below the figure.

We also investigate the relationship between structural network motifs and biological network motifs in this work. Table 5 is the table generated by FANMOD [9] to observe the statistical properties of each 4-node subgraph type in the DIP core network. The first column is the label for each type generated by *Nauty *program [24] and Figure 1 shows shape for each label of subgraph. Second column indicates the percentage of each type appears in the DIP Core network and the next two columns show the average frequencies and standard deviation of each type, out of 10, 000 randomized graphs. Last two columns of Z-score and P-value show the structural statistics of each type. As a subgraph type of Z-score larger than 2.0 or P-value smaller than 0.01 is a network motif, in DIP Core network, the five types of C^, CN, CF, C~, and Cr are network motifs. Figure 2 shows relative frequencies for each subgraph types, where the horizonal axis lists all six types and vertical axis indicates its relative frequency. Each line refers to a result of each algorithm, differentiated by colors. All of the algorithms except ESU reduce the total number of subgraph search to 30%, but the relative frequencies are almost same as those of ESU. In fact, when we plug each of the reduced network, which is the by-product of each algorithm, in FANMOD, the same five types of subgraphs ( C^, CN, CF, C~, Cr) are detected as network motifs. It proves that our algorithms are applicable to find structural network motifs as well, but more efficiently.

**Table 5:** DIP Core- statistical properties, from FANMOD

Label	Freq(Original)	Mean-Freq (Random)	S-Dev(Random)	Z-score	P-value
**C^**	1.46%	5.9e-005%	3.04e-006	4813.3	< 10^-3^

CN	10.21%	0.01%	< 10^-6^	289.09	< 10^-3^

CF	48.69%	42.22%	< 10^-6^	17.31	< 10^-3^

**C~**	0.48%	0.00%	0	undefined	< 10^-3^

Cr	0.47%	0.23%	< 10^-6^	16.28	< 10^-3^

CR	38.65%	57.54%	< 10^-6^	-52.17	> 10^-2^

Each type of 4-node subgraph shows its significance based on its structural uniqueness. The label is generated by *Nauty *program [24] and the corresponding shape is shown in Figure 1. All types except CR are structural network motifs by definition.

**Figure 3:**
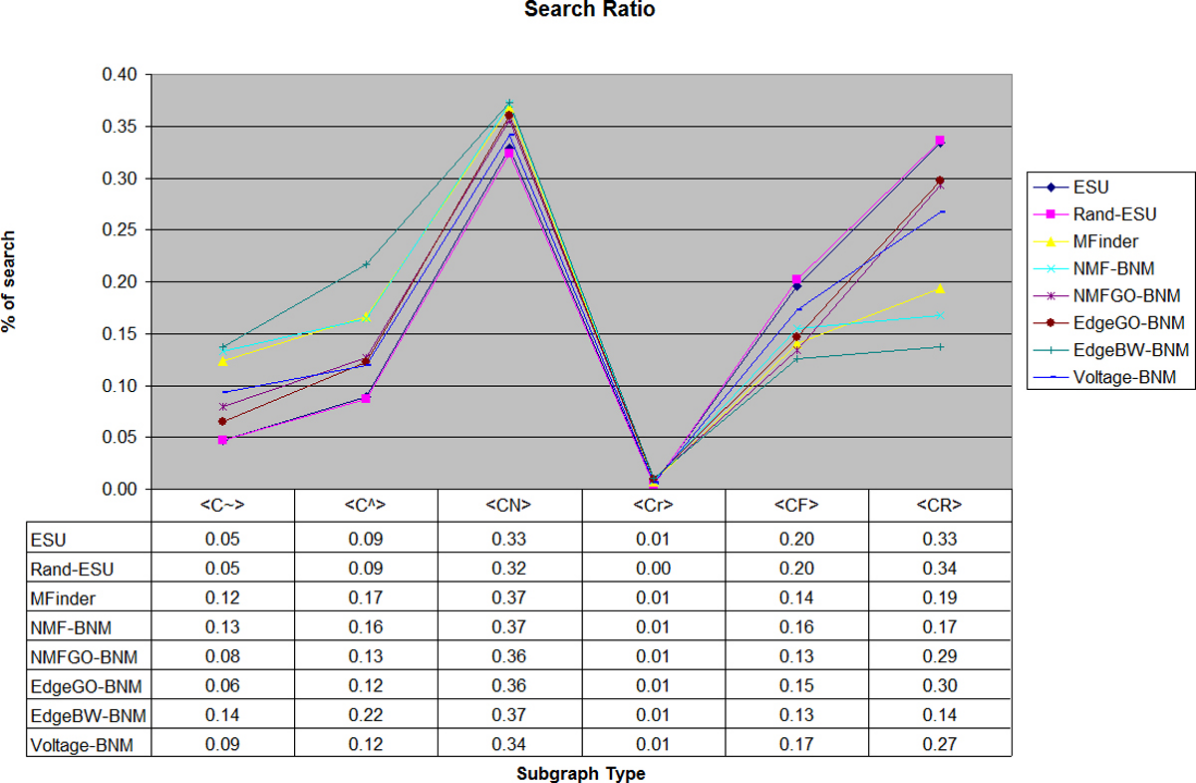
**Y2k network: Search ratios based on the subgraph type**. The ratio of frequency of each type is relatively preserved and it indicates that our algorithms can be used for the structural network motif discovery as well. The description of the plots and the table is same as in Figure 2.

We analyze Y2k network as well to see a relevance of structural network motif and biological network motif. Table 6 generated by FANMOD identifies top three subgraphs as network motifs, labeled C~, C^ and CN. Similar to DIP core network, all of the algorithms preserve relative frequencies for each type as appeared in Figure 3 and FANMOD confirms that the same three types are still the structural network motifs in the reduced Y2k networks as well.

**Table 6:** Y2k- statistical properties, from FANMOD

Label	Freq(Original)	Mean-Freq (Random)	S-Dev(Random)	Z-score	P-value
**C~**	4.66%	4.07e-006%	9.14e-007	51013	< 10^-3^

C^	8.91%	< 10^-2^	4.29e-005	2075.1	< 10^-3^

CN	32.89%	0.021%	< 10^-6^	225.64	< 10^-3^

Cr	0.55%	1.14%	< 10^-6^	-9.95	> 10^-2^

CF	19.58%	41.82%	< 10^-6^	-66.188	> 10^-2^

CR	33.40%	57.06%	< 10^-6^	-84.16	> 10^-2^

Each type of 4-node subgraph shows its significance based on its structural uniqueness. The label is generated by *Nauty *program [24] and the corresponding shape is shown in Figure 1. In this network, the first three types are detected as network motifs.

### Biological significance for biological network motifs

We provide one example which demonstrates that EDGEGO-BNM is especially good for discovering biological network motifs included in protein functional modules. This example also shows that structurally non-motifs cannot be ignored as many of the instances have biological significance. Table 7 shows the recall value of 4-node biological network motifs included in a 'rRNA processing' functional module in yeast, based on different subgraph type and algorithms. We exactly count the numbers of motifs included in 'rRNA processing' with ESU algorithm first. Then all other algorithms are compared with the recall in Equation (1).

**Table 7:** Y2k network: the rates of motifs included in a 'rRNA processing' functional module in yeast, computed using equation (1).

Algorithm	C~	C^	CN	Cr	CF	CR
**ESU (Counts)**	**1.0(2,509)**	**1.0(5,152)**	**1.0(17,457)**	**1.0(434)**	**1.0(8,095)**	**1.0(15,953)**
RAND-ESU	.30	.32	.34	.36	.34	.34
MFINDER	.78	.54	.31	.38	.16	.13
**EDGEGO-BNM**	.97	.97	.98	1.0	.99	.97
EDGEBETWEENNESS-BNM	.67	.64	.32	.57	.22	.16
NMFGO-BNM	.87	.88	.78	.89	.70	.73
NMF-BNM	.69	.39	.23	.22	.12	.90
VOLTAGE-BNM	.53	.38	.39	.39	.32	.31

Except ESU, all algorithms only search 30% of subgraphs in the original network. However, EDGEGO-BNM recovers over 90% of motifs included in functional module. We note that the non-motif types of Cr, CF and CR have a number of instances for this functional match, indicating structural uniqueness is insufficient to discover its biological significance.

(1) $$\text{Recall = }\frac{\text{discovered number of motifs included in a ’rRNA processing’ }\text{with the algorithm}}{\text{true number of motifs included in a rRNA processing}}$$

In Table 7, the first column lists all the algorithms conducted in this paper, and the other columns show the recall of subgraphs included in 'rRNA processing' functional module according to each subgraph type. The 'rRNA processing' functional module consists of 206 proteins in the yeast. All algorithms except ESU search only 30% of subgraphs out of the total subgraphs searched with ESU algorithm but EDGEGO-BNM recovers over 90% of subgraphs included in 'rRNA processing'. Furthermore, we observe that although the Cr, CF, CR are structural network non-motifs, about 50% of subgraphs included into the 'rRNA processing' are these non-motifs. This example shows that even non-motifs also have biological meanings, therefore the structural network motif defined by its structural uniqueness is insufficient to explain biological meanings.

## Conclusions

In this paper, we provide new approaches to finding network motifs in biological networks. Structural network motifs are defined as frequently and uniquely repeated small connected subgraph in a network. However, motivated by several issues brought up while a number of network motif applications are investigated, we propose to find biologically meaningful network motifs. Hence, we define **biological network motifs **as biologically meaningful *k*-node subgraphs, develop a number of algorithms for efficient detection of biological network motifs and introduce new evaluation measures. The algorithms reduce the number of subgraph search and increase the detection rates of biological network motifs at the same time. The algorithms are categorized into two classes: Edge-removing algorithms and Network clustering algorithms. EDGEGO-BNM and EDGEBETWEENNESS-BNM are algorithms which remove a number of edges based on GO term and edge betweenness score, respectively. NMF-BNM, NMFGO-BNM and VOLTAGE-BNM algorithms partition the network based on its topological property or GO term relevance. All the algorithms introduced in this paper improve existing algorithms for high quality structural network motif detection.

We also introduce a number of evaluation measures which measure biological significance of each subgraph: 'motifs included in complex', 'motifs included in functional module' and 'GO term clustering score.' Biological meanings of those biological network motifs are assigned based on these evaluation measures. We ran the algorithms on two PPI network of *S. cerevisiae*, and compared them with our new measures. An existing exhaustive search and other two existing approximation algorithms are also provided to be compared with our algorithms. EDGEGO-BNM shows overall good results in all the measures, but EDGEBETWEENNESS-BNM is the best in the 'motifs included in complex' measure.

The works in this paper can be studied further. Currently, the parameters of various algorithms in this paper are adjusted only to obtain a desired number of subgraphs. In near future, various impacts of the parameters on the results should be investigated. Besides the parameters, the balance between topological and biological information will be an important factor for a better algorithm. On the other hand, current evaluation measures are limited to PPI networks. Comprehensive evaluation measures should be designed to apply various types of biological networks. Meanwhile, the work should be extended to weighted or direct networks for more comprehensive analysis of biological network motifs.

## Methods

### Definitions and notations

We assume that a biological network is a graph *G *= (*V*, *E*) where each vertex in *V *is a molecule and each edge in *E *is an interaction between vertices. A **network motif ***m *is a connected subgraph of size *k *in a graph, which appears more frequently than usual. The size of network motif, *k*, ranges from 3 up to 15 or more, but relatively very smaller than the number of vertices in the network, |*V*|. The frequency *f*_*G*_(*m*) of *m *is the number of isomorphic graphs to *m *in *G*. To determine the uniqueness of *m*, a number of random graphs, typically more than 10,000 graphs, are generated and the frequencies *f*_*R*_(*m*) is recorded for each generated graph *R *to obtain a P-value as in Equation (2) or a Z-score in Equation (3).

(2) $$P\left(m\right)=\frac{1}{N}\overset{N}{\underset{n=1}{\sum }}c\left(n\right),\;\text{where}\;c\left(n\right)=\left\{\begin{array}{cc} 1, & \text{if}\;f_{R}\left(m\right)\geq f_{G}\left(m\right);\\ 0, & \text{otherwise}. \end{array}\right)$$

(3) $$Z\left(m\right)=\frac{f_{G}\left(m\right)-average\left(f_{R}\left(m\right)\right)}{std\left(f_{R}\left(m\right)\right)}$$

Here *average*(*f*_*R*_(*m*)) and *std*(*f*_*R*_(*m*)) refer to the average and standard deviation of frequencies in random networks respectively. Generally, a subgraph *m *with P-value less than 0.01 or Z-score greater than 2.0 is considered as a network motif.

We define a **biological network motif ***g *as a small connected subgraph of size *k *which has topological property as well as biological meanings. For clear understanding, a network motif is referred to **structural network motif **throughout this paper, and biological network motifs and structural network motifs have many-to-many relationships. We emphasize that we do not categorize all of the biological network motifs into some classes like 'motif mode' in the study by Lee and Tzou [25], where the number of motif modes reaches up to millions. Instead, we assume that biological network motifs are application dependent, therefore flexibly categorized according to the applications. For a specific subgraph being a biological network motif, we need some measures which are presented later in this section. From now on, *G *= (*V*, *E*) is a target (original) network, *G' *= (*V*, *E'*) is a modified network, *n *is the number of vertices and *m *is the number of edges in *G*.

### Description of Algorithm

Structural network motifs are either exactly (exhaustively) or approximately determined. As exhaustive search is infeasible in large networks, approximation algorithms have been used in many applications in practice. In this study, we provide a number of algorithms originally designed to detect biological network motifs, but also improve existing algorithms for high quality structural network motif discovery. Some algorithms use structural information alone or biological information alone, and others combine structural and biological information.

**Figure 4:**
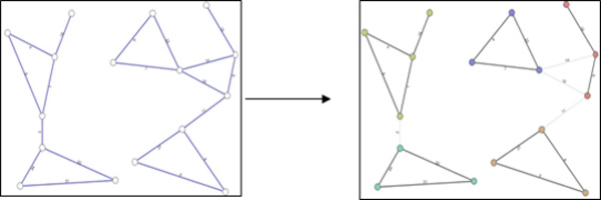
**After graph modify**. Original network (left) and the modified network (right) after removing edges or clustering the graph, where a number of clusters and a list of removed edges are provided as a result.

The main idea of the algorithms is to modify the original network so that we can increase the biological network motif detection rates over total number of subgraphs in the original graph. For example, if we remove 23% of edges, then the number of subgraphs are reduced to 30% of the total number. We provide two ways of modifying the original network: 1) removing a number of edges and 2) clustering the network into smaller sub-networks. The two measures provide essentially the same components, a list of removed edges and a number of clusters as shown in Figure 4. When we remove some edges, we obtain a number of clusters as by-products. When we cluster a network, the edges in between clusters will be listed in the set of removed edges.

#### Edge-Removing Algorithms

We present two algorithms to remove 'insignificant' edges based on two different aspects. EDGEGO-BNM (EDGEGO for biological network motif) algorithm removes edges based on its related Gene ontology terms. EDGEBETWEENNESS-BNM (EDGEBETWEENNESS for biological network motif) algorithm removes edges based on its edge betweenness score. Since EDGEGO-BNM algorithm uses Gene ontology (GO) terms associated with the nodes, the algorithm is applicable only to the gene or protein related networks. In EDGEBETWEENNESS-BNM algorithm, although the computation of EDGEBETWEENNESS score is existing measure used for network clustering [26], it is the first time used for network motif detection.

##### EDGEGO-BNM algorithm

**Figure 5:**
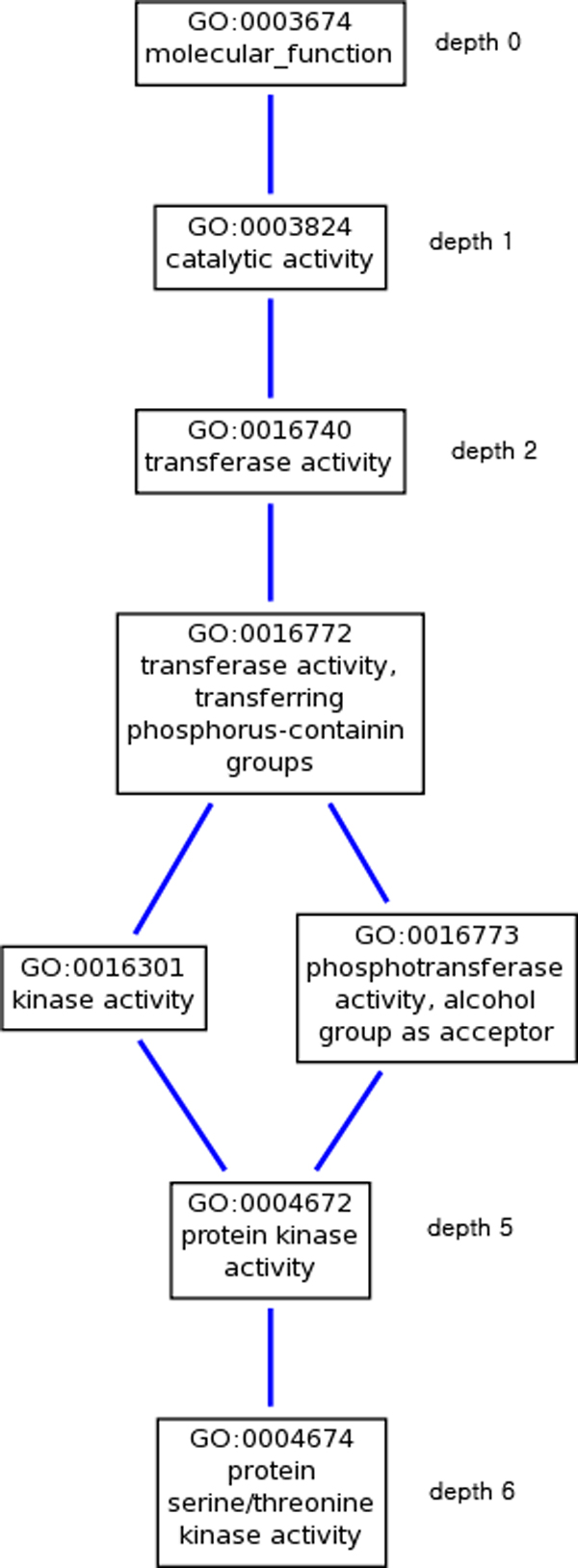
**GO DAG example**. GO DAG example view, where the root node is a molecular function (MF) GO term.

In this algorithm, we reduce the number of subgraphs to be searched by removing a number of 'biologically insignificant' edges in the original network. Biologically insignificant edges are determined with the Gene ontology (GO) [27] terms associated with its end points. GO terms provide annotations of gene and gene product attributes across species and databases. GO consists of three independent domains: biological process (BP), molecular function (MF) and cellular component (CC). A BP refers to series of events by multiple molecular functions. Examples include cellular physiological process and pyrimidine metabolic process. An MF is a molecular level of activities, such as catalytic activity or binding. A CC is a component of a cell which is part of larger item. Examples are nucleus, ribosome or proteasome. With the three orthogonal aspects as roots, GO is represented as a directed acyclic graph (DAG), a part of which is shown in Figure 5. GO DAG describes each GO term as a node and the relationships as an directed edge with hierarchical structure, where children are more specific than the parents. Each term can have multiple parents as well as multiple children and it is traced backward to the root of depth 0. If a gene *ge *is annotated with a GO term *pe*, then *ge *is annotated with all of the ancestor GO terms of *pe*.

We define an **EdgeGO set **as a set of all GO's associated to both of the end points of the edge *e *and an **EdgeGO depth of ***e *is the maximum depth of the GOs in the EdgeGO set. In EDGEGO-BNM algorithm, a threshold GO term depth *d *should be given as a parameter and the edges whose EdgeGO depth is less than *d *are removed. Algorithm 1 describes detail steps of the EDGEGO-BNM algorithm.

**Algorithm 1**: EDGEGO-BNM

**input**: Graph *G *= (*V*, *E*), *d *:a GO depth threshold, *k *:the motif size.

**output**: a number of subgraphs with size *k*.

1 *RE *← ∅

2 *E' *← *E*

3 **for **∀*e *∈ *E ***do**

4   *GO set *← all GO terms associated with both of the endpoints of *e*

5   *D *← maximum depth of *GOset*

6   **if ***D *<*d ***then**

7      *RE *= *RE *∪ {*e*}

8      *E' *= *E' *- {*e*}

9 Let *G' *= (*V*, *E'*)

10 Enumerate all k-subgraphs from *G'*

Line 10 in Algorithm 1 produces all the *k*-size subgraphs in the reduced graph *G'*, and any existing exact counting algorithm can be used for this task. In EDGEGO-BNM algorithm, different depth threshold *d *results different number of edges to remove and we experimentally determine the threshold depth to get a desired number of subgraphs. More edges are removed as the depth threshold increases, which in turn reduces the number of subgraph searches. This work is motivated by the paper [20] which reveals that different levels of GO terms lead to different modes of motifs. EDGEGO-BNM algorithm is deterministic and the whole process except line 10 runs linearly with the number of edges, *m*. In most cases, this algorithm obtains unbalanced clusters, where a few clusters have most of the vertices and most of the clusters consist of small number of vertices.

##### EDGEBETWEENNESS-BNM algorithm

EDGEBETWEENNESS-BNM algorithm uses topological information to remove some of edges. EDGEBETWEENNESS algorithm is initially introduced by Girvan and Newman [26] to produce network clusters using betweenness score of each edge. Network modularization [28] is supported by this measure and many protein modules are successfully discovered with it. EDGEBETWEENNESS-BNM algorithm goes through all edges to compute its edge betweenness score, namely, *EBScore*: The number of shortest paths in all pairs of vertices that run along with the edge e is *EBScore*(*e*), then the edge with the highest *EBScore *is removed. This process is repeated until we get a desired number of edges to remove. The detail procedure of EDGEBETWEENNESS-BNM is described in Algorithm 2.

**Algorithm 2**: EDGEBETWEENNESS-BNM

**input **: Graph *G *= (*V*, *E*), *r *is the number of edges to remove, *k *:the motif size.

**output**: a number of subgraphs with size *k*.

1 *RE *← ∅

2 *E' *← E

3 *R *← 0

4 **while ***R < r ***do**

5   for all pairs of vertices in *V*, obtain the shortest path, *SP*

6   ∀*e *∈ *E*, let *EBscore*(*e*) = number of SP's containing *e *in the path

7   Let *ed *be the edge with maximum *EBscore*

8   *RE *= *RE *∪ {*ed*}

9   *E' *= *E' *- {*ed*}

10   *R *= *R *+ 1

11 Let *G' *= (*V*, *E'*)

12 Enumerate all k-subgraphs from *G'*

Except line 12 in Algorithm 2, EDGEBETWEENNESS-BNM algorithm runs in *O*(*rmn*) where *r *is the number of edges to remove. EDGEBETWEENNESS-BNM algorithm produces relatively balanced network clusters and is also a deterministic algorithm.

#### Clustering Algorithms

Another way of reducing a network is to partition the network into smaller sub-networks and remove the edges between clusters. In this work, we present three clustering algorithms: NMF-BNM (Nonnegative matrix factorization for biological network motif), NMFGO-BNM (Nonnegative matrix factorization with GO term for biological network motif) and VOLTAGE-bnm(Voltage clustering for biological network motif) algorithm. Voltage clustering algorithm has been used for network clustering before, but not for network motif discovery.

##### NMF-BNM algorithm

Nonnegative matrix factorization (NMF) has been used to cluster various data, such as face images, text corpus and gene expression data. Initially used as a dimension reduction technique, NMF is successfully applied to many clustering tasks with additional sparseness constraints [29-31]. In this work, we apply NMF for an efficient detection of biological network motif. Detail process of NMF-BNM is described in Algorithm 3.

**Algorithm 3**: NMF(GO)-bnm

**input **: Graph *G *= (*V*, *E*), *c *is the number clusters, *k *:the motif size, (*d *is GO depth threshold), *η*

and *β *for sparse NMF.

**output**: a number of subgraphs with size *k*.

1 *RE *← ∅

2 *E' *← *E*

3 Let *CL*_1_, ⋯, *CL*_*c *_= ∅.

4 Construct a data matrix A from *G*.

5 Run sparse NMF to *A *and get an *n *× *c *matrix *H*

6 **for ***all the columns in H ***do**

7   Let $h^{j}={\left\{h_{1}^{j},\cdot \cdot \cdot ,h_{c}^{j}\right\}}^{T}$ be *j*th column vector of *H*.

8   **if **$h_{i}^{j}$*is largest in h*^*j *^**then**

9      put the vertex *v*_*j *_to *CL*_*i*_.

10 **for **∀*e *∈ *E ***do**

11   **if ***e lies between clusters of CL*_*i *_**then**

12      RE = *RE *∪ {*e*}

13      *E' *= *E' *- {*e*}

14 Let *G' *= (*V*, *E'*)

15 Enumerate all k-subgraphs from *G'*

In NMF-BNM, a nonnegative matrix *A *= (*a*_*ij*_) of line 4 in Algorithm 3 is topology-based feature data as shown in Equation (4) and sparseness constraints are added for better clustering. In sparse nonnegative matrix factorization (Algorithm 3 line 5), the data matrix *A *are decomposed into two factor matrices *W *and *H *using the objective function in Equation (5).

(4) $$a_{ij}=\frac{1}{|v_{i}-v_{j}{|}^{2}},1\leq i,j,\leq n$$

(5) $$\underset{W,H}{\min}\frac{1}{2}\left\{||A-WH|{|}_{F}^{2}+\eta ||W|{|}_{F}^{2}+\beta \overset{m}{\underset{j=1}{\sum }}||H\left(:,j\right)|{|}_{1}^{2}\right\}\text{subject to }W\geq 0,H\geq 0.$$

Here, $||.|{|}_{F}^{2}$ is the square of the Frobenius norm, $||.|{|}_{1}^{2}$ of the *L*_1 _norm, and *H*(:,*j*) is the *j*th column of matrix *H*. Two parameters, *η *for sparseness and *β *for balance between sparseness and correctness, should be given. Intuitively, the matrix *H *gives clustering information as described in lines 6 to 9. The detail description of sparse NMF is illustrated in the paper [31] by Kim and Park. Except the last step in Algorithm 3, NMF-BNM runs linearly with the size of *A *at each iteration, and it converges to a stable point, not necessarily unique, through a number of iterations.

##### NMFGO-BNM algorithm

NMFGO-BNM algorithm differs from NMF-BNM only in line 4 of Algorithm 3, where the feature matrix *A *= (*a*_*ij*_) combines structural and GO term information of the network as shown in Equation (6). In this algorithm, an additional parameter *d*, which is a GO term depth threshold, is given. First, all the GO terms associated with the network and whose depth is greater than *d *are listed. Suppose the list of GO terms is {*g*_1_, *g*_2_, ⋯, *g*_*p*_}, then each entry *a*_*ij *_in the (*n *+ *p*) × *n *matrix *A *is defined as in Equation (6). The rest of process is the same as of the NMF-BNM algorithm.

(6) $$\begin{array}{llll} a_{ij} & =\frac{1}{|v_{i}-v_{j}{|}^{2}},\text{if }1\leq i,j\leq n\; & & \;\\ & =1,\text{if }v_{j}\text{ is annotated with }g_{i-n}\text{ and }n<i\leq \left(n+p\right),1\leq j\leq n\; & & \;\\ & =0\text{ if }v_{j}\text{ is not annotated with }g_{i-n}\text{ and }n<i\leq \left(n+p\right),1\leq j\leq n\; & & \;\\ & \; & \end{array}$$

##### VOLTAGE-BNM algorithm

VOLTAGE clustering algorithm is developed by Wu and Huberman [32] to cluster a network based on voltage drops. The algorithm first generates a number of candidate clusters using Kirchhoff equations [33], which tell that total current of each node should sum up to zero. From the candidate clusters, a seed is selected which appears most frequently in the candidate clusters, and the neighbor vertices of this seed are collected to form a cluster. The process is repeated until we get a desired number of clusters. The number of clusters are later adjusted if the seeds are too close. An exact solution for this algorithm requires O(|*V*|^3^), but Wu and Huberman [32] provide an approximation solution in *O*(|*V*| + |*E*|). In this paper, we utilize VOLTAGE clustering algorithm to design a VOLTAGE-BNM (voltage for biological network motif) algorithm for efficient discovery of biological network motifs as shown in Algorithm 4. We emphasize that VOLTAGE-BNM algorithm is easy and fast, but it is non-deterministic algorithm because the randomly selected seeds lead to quite different results every time it runs.

Algorithm 4: VOLTAGE-BNM

**input **: Graph *G *= (*V*, *E*), *c *is the number clusters, *k *:the motif size.

**output**: a number of subgraphs with size *k*.

1 *RE *← ∅

*2 E' *← *E*

3 Let *CL*_1_, ⋯, *CL*_*c *_= ∅.

4 *m *← **0**.

5 **while *(****m *≤ *c*) **do**

      //Generate *c *number of candidate clusters.

6   Pick a vertex pair, *source *and *sink*.

7   Compute voltages of each vertex of graph *G *using *source *and *sink*.

8   Group the vertices in two clusters (high/low).

9   Store resulting candidate clusters.

10   *m *= *m *+ 2

11 *l *← 1

12 **while ***l *<*c ***do**

      //generate*c *- 1 clusters

13   Pick one cluster seed *s *most appearing in candidate clusters.

14   Obtain co-occurrence vertices to the s, and put them to a cluster *CL*_*l*_.

15   Remove all the co-occurrence vertices and *s *from candidate clusters.

16   *l *= *l *+ 1.

17   Remaining unassigned vertices belong to the *CL*_*c *_cluster.

18 if ∀*e *∈ *E*, *e lies between clusters of CL*_i_, **then**

19   *RE *= *RE *∪ {*e*}

20   *E' *= *E' *- {*e*}

21 Let *G' *= (*V*, *E'*)

22 Enumerate all k-subgraphs from G

Table 8 summarizes the algorithms introduced in this paper. As all of the algorithms have a common step of 'Enumerate all *k*-subgraphs from *G'*, the time in this table excludes this last step.

**Table 8:** Various algorithms used for the detection of biological network motifs

Algorithm	Type	Time before ESU	Parameter	Deterministic
EDGEGO-BNM	Edge-Removing	*O*(|*E*|)	*d*	Yes
EDGEBETWEENNESS-BNM	Edge-Removing	*O*(*r*|*E*||*V*|)	*r*	Yes
NMFGO-BNM	Clustering	*O*(|*E*|(|*V*| + *l*))	*d*, *c*, *η*, *β*	No
NMF-BNM	Clustering	*O*(|*E*||*V*|)	*c*, *η*, *β*	No
VOLTAGE-BNM	Clustering	*O*(|*E*| + |*V*|)	*c*	No

All the algorithms introduced in this paper are compared based on type, time before enumeration, parameter, and whether its deterministic property. Here *d *is GO depth threshold,*l *is the number of GO terms associated to the graph *G*, *c *is the number of clusters, *r *is the number of edges to remove, and *η*, *β *for sparse NMF computation.

### Evaluation Measures

Network motif is defined as a frequently and uniquely represented subgraph in a network and is determined through structural uniqueness, measured by P-value (9) or Z-score (3). The structural uniqueness, however, is an inappropriate validation for motifs in biological networks. Therefore, we design several biological evaluation measures other than topological uniqueness in this study. These are called 'motifs included in complex', 'motifs included in functional module', 'GO (Gene ontology) term clustering score'. Protein complexes are the groups of proteins interacting with each other at the same time and same place in a cell, whereas functional modules are the groups of proteins binding to participate in different cellular processes at different times. Currently, these evaluation measures are specifically designed for PPI networks. More comprehensive validation measures should be developed in near future.

#### Motifs included in complex

The first assessment is a match with a protein complex. We consider a subgraph *g *is **included in a complex **if a known protein complex contains all the nodes in *g*. We define ***motif included in complex ***measure as the precision of the subgraphs included in protein complexes as shown in Equation (7). Obviously, the algorithm with higher value for this measure performs better in this work.

(7) $$\text{Motifs included in complex = }\frac{\text{number of motifs included in a complex}}{\text{number of all discovered subgraphs}}$$

#### Motifs included in functional module

Similar to the previous measure, if all components of a subgraph *g *are included in a known protein functional module, *g *is **included in a functional module**. Therefore ***motif included in a functional module ***is defined as the precision of the subgraphs included in functional modules as in Equation (8).

(8) $$\text{Motifs included in functional module = }\frac{\text{number of motifs included in a functional module}}{\text{number of all discovered subgraphs}}$$

In our experiments, the database for protein complexes and functional modules are obtained from MIPS [34] server.

#### GO term clustering score

We define a **P-value of a subgraph g **as the minimum P-value over the union of GO terms of *g *and lower P-value is preferable. P-value for a GO term is computed using hypergeometric distribution as in Equation (9), where *N *is the whole population, *M *is the population that is annotated by the GO term, *n *is the subgraph size and *x *is the number of genes annotated with the GO term in the sample.

(9) $$P-value=\sum_{j=x}^{n}\frac{\left(\begin{array}{c} M\\ j \end{array}\right)\left(\begin{array}{c} N-M\\ n-j \end{array}\right)}{\left(\begin{array}{c} N\\ n \end{array}\right)}$$

To determine if a subgraph *g *with a P-value *p *is significant, a cutoff value should be pre-defined. Since P-value decreases as the size of *g *increases, higher cutoff value is necessary for small-size of subgraph *g*. For 4-node and 5-node subgraph, we set the cutoff value as 0.1 and if the P-value of *g *is lower than the cutoff, *g *is a *significant *subgraph. A better algorithm should provide more significant subgraphs and lower average p-value of the subgraphs. In other words, average P-value alone, or the number of significant subgraphs alone cannot fairly assess the performance of an algorithm. To evaluate the overall performance of an algorithm, we use the ***clustering score ***introduced in the studies of [28,35] which has measured the quality of clustering algorithms. For a ***GO term clustering score ***measure, we use subgraphs instead of clusters in the formula (10),

(10) $$\text{clustering score}=1-\frac{\sum_{i=1}^{n_{s}}min\left(pi\right)+\left(n_{i}\cdot \text{cutoff}\right)}{\left(n_{s}+n_{i}\right)\cdot \text{cutoff}},$$

where *min*(*pi*) is the P-value of each subgraph, *n*_*s *_is the number of significant and *n*_*i *_is the number of insignificant subgraph. A higher GO term clustering score of an algorithm indicates a better algorithm. Since GO term has three independent aspects of BP, MF, CC, we have three types of this measure: BP GO term clustering score; MF GO term clustering score; and CC GO term clustering score.

## List of abbreviations

BNM: Biological Network Motif; GO: Gene Ontology; BP: Biological Process; MF: Molecular Function; CC: Cellular Component; DAG: Directed Acyclic Graph; SP: Shortest Path; NMF: Non-negative Matrix Factorization; ERS: Exhaustive Recursive Search; ESU: Enumerate SUbgraph; RAND-ESU: Randomized ESU.

## Competing interests

The authors declare that they have no competing interests.

## Authors' contributions

The research was conceived and planned by WK, ML and YP. WK designed the algorithms and new evaluation measures. The experiments were performed and analyzed by WK, ML and YP. YP and JW supervised the work and helped to draft the manuscript. All authors read and approved the final manuscript.
